# Effects of Vacuum Annealing on the Conduction Characteristics of ZnO Nanosheets

**DOI:** 10.1186/s11671-015-1066-1

**Published:** 2015-09-17

**Authors:** Chris J. Barnett, Nathan A. Smith, Daniel R. Jones, Thierry G. G. Maffeis, Richard J. Cobley

**Affiliations:** Multidisciplinary Nanotechnology Centre, College of Engineering, Swansea University, Singleton Park, Swansea, SA2 8PP UK; Department of Physics, College of Science, Swansea University, Singleton Park, Swansea, SA2 8PP UK

**Keywords:** ZnO, LBZA, Nanosheets, Defects, Resistance, 81.07.-b, 62.23.Kn, 73.63.Rt

## Abstract

ZnO nanosheets are a relatively new form of nanostructure and have demonstrated potential as gas-sensing devices and dye sensitised solar cells. For integration into other devices, and when used as gas sensors, the nanosheets are often heated. Here we study the effect of vacuum annealing on the electrical transport properties of ZnO nanosheets in order to understand the role of heating in device fabrication. A low cost, mass production method has been used for synthesis and characterisation is achieved using scanning electron microscopy (SEM), photoluminescence (PL), auger electron spectroscopy (AES) and nanoscale two-point probe. Before annealing, the measured nanosheet resistance displayed a non-linear increase with probe separation, attributed to surface contamination. Annealing to 300 °C removed this contamination giving a resistance drop, linear probe spacing dependence, increased grain size and a reduction in the number of n-type defects. Further annealing to 500 °C caused the n-type defect concentration to reduce further with a corresponding increase in nanosheet resistance not compensated by any further sintering. At 700 °C, the nanosheets partially disintegrated and the resistance increased and became less linear with probe separation. These effects need to be taken into account when using ZnO nanosheets in devices that require an annealing stage during fabrication or heating during use.

**Electronic supplementary material:**

The online version of this article (doi:10.1186/s11671-015-1066-1) contains supplementary material, which is available to authorized users.

## Background

ZnO nanomaterials have received much attention over the past 15 years due to their novel properties including being a wide band gap (3.37 eV) piezoelectric material with a large exciton binding energy of 60 meV [1, 2]. ZnO nanomaterials have many potential applications including antimicrobial bio-films [3], microelectronics [4], mechanical energy harvesting [5], field emitters [6], ultra violet lasers [7], photovoltaics [8] and other optoelectronic devices [9]. Polycrystalline ZnO nanosheets are a relatively new form of nanostructure and have demonstrated promising potential for practical applications such as gas-sensing devices and dye sensitised solar cells due to their high surface area to volume ratio [10, 11].

In order to study the intrinsic properties of materials, vacuum annealing is commonly used to remove surface contamination [12]. Furthermore, standard semiconductor fabrication techniques often include annealing stages for dopant activation, Ohmic contact formation and implantation repairs [12]. Additionally, devices including gas sensors operate at high temperature which can alter the grain structure of polycrystalline materials [13]. Our earlier work found that grains within the nanosheets start to sinter when annealed at 700 °C in air [14]. Also, photoluminescence (PL) found that the shape and size of the deep level emission (DLE) peak indicated a significant relative increase in p-type defects with annealing temperature, attributed to a decrease in oxygen vacancies. Oxygen vacancies give rise to the inherent n-type nature of ZnO and therefore any changes in these vacancies due to annealing suggest a change in the resistivity of the nanosheets [15]. However, the evolution of transport properties through ZnO nanosheets after annealing has not been studied.

Here, we investigate the effects of vacuum annealing on the chemical composition, morphology, optical and conductive properties of ZnO nanosheets using nanoscale two-point probe, Auger electron spectroscopy (AES), PL and scanning electron microscopy (SEM).

## Methods

Layered basic zinc acetate (LBZA) nanosheets were synthesised using our previous method [14]. A 500 ml solution of 0.1 M zinc acetate dihydrate (Zn(CH_3_COO)_2_.2H_2_O) and 0.04 M hexamethylenetetramine (HMTA, (CH_2_)_6_N_4_) from Sigma Aldrich Co. Ltd was heated in an 800 W commercial microwave for 6 min. The solution was centrifuged, the supernatant removed and the residue re-suspended in DI water. The resulting nanosheets were deposited onto silicon with 100 nm of thermal oxide (referred to as Sample 1) and two pieces of n-type (100) silicon from the Institute of Electronic Materials Technology (referred to as Sample 2 and Sample 3), and all three samples were initially annealed in air at 400 °C to thermally decompose the LBZA to ZnO.

Two-point probe measurements were carried out on Sample 1 in ultra-high vacuum (UHV) using an Omicron LT Nanoprobe equipped with a NanoSAM electron analyser, base pressure 1 × 10^−10^ mbar. Sample 2 was characterised with AES within the Nanoprobe chamber, and Sample 3 with high-resolution SEM using a Hitachi S4800 and PL using a 325-nm wavelength He-Cd laser and an Ocean Optics USB2000+ spectrometer. All characterisation techniques were carried out at room temperature. All samples were annealed to 300, 500 and 700 °C for 1 h in UHV and allowed to cool before being re-characterised. Sample 1 and Sample 2 were not removed from the vacuum between heating stages and analysis. For the PL, three spectra were taken from different areas of the sample and averaged. The AES was performed at 20K magnification using a beam acceleration voltage of 5 kV and 1 nA beam current through a 90 μm beam aperture.

**Fig. 1:**
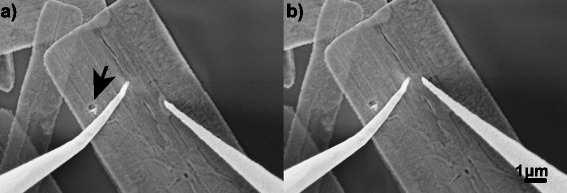
**a** First position of the tungsten probes on a ZnO nanosheet with an *arrow* marking a dislocation and **b** final position of the tungsten probes on the ZnO nanosheet

Two-point probe measurements were carried out using tungsten probes, annealed to reduce probe oxide contamination [16, 17]. Two tungsten probes were approached using a method developed to ensure minimal compressive strain at the point of contact providing intrinsic characterisation of the nanosheet [18]. *I-V* sweeps were performed from −1 to 1 V at five probe separations with two of the positions shown in Fig. 1, each repeated five times. After annealing the nanosheets in vacuum, the probes were repositioned onto the same measurement locations, on the same nanosheet, as shown in the Additional file 1: Figure S1.

## Results and Discussion

**Fig. 2:**
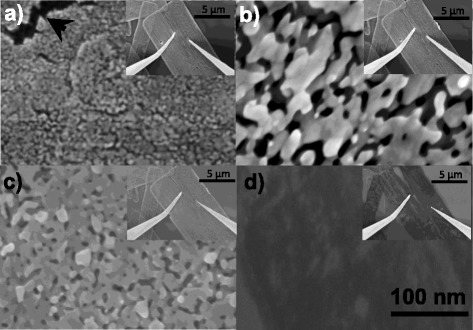
SEM images of ZnO nanosheets (**a**) before vacuum annealing with an *arrow* marking a void and after vacuum annealing at **b** 300 °C, **c** 500 °C and **d** 700 °C, with insets showing probe positions

The synthesised nanosheets were of rectangular form with width typically ranging from 3 to 12 μm, lengths typically ranging from 8 to 25 μm and thicknesses typically ranging from 20 to 100 nm. Figure 2 shows SEM images of the grain structure before and after vacuum annealing. Before vacuum annealing the average grain size was 5 ± 1.3 nm, as measured from one representative high-resolution SEM frame. After annealing to 300 °C, the grains sintered to form clusters of around 50–100 nm. The sintered grain size was much larger than that measured following similar annealing in air and is more comparable to annealing at 800 °C in air [14]. This is likely due to the effect of the melting point dependence on pressure, which is well-documented [19, 20]. At 500 °C, the grains sintered further, whilst at 700 °C the nanosheets appear much darker, as shown by the SEM image in Fig. 2d indicating lower conductivity, and show signs of fracturing and partial disintegration.

**Fig. 3:**
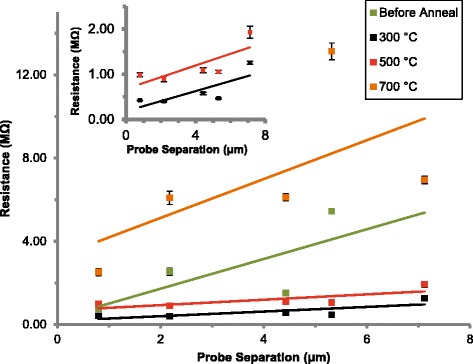
Mean two-point probe resistance measurements at +1 V with standard deviation, against probe separation for ZnO nanosheets before and after vacuum annealing at 300, 500 and 700 °C, with *inset* showing 300 and 500 °C. A least squares linear fit is overlaid for all

In Fig. 3, the mean nanosheet resistance at +1 V measured using the two-point probe is shown against probe separation. One outlier with a standard deviation more than 12 times the average was removed. For homogenous materials, a standard resistivity model would give a linear increase with probe separation. For polycrystalline materials, the resistance primarily increases in a step-like fashion across the grain boundaries [21]. However, since the grain size here is much smaller than the length scale shown, the trend should still be linear. Therefore, perturbations away from linearity in the data in Fig. 3 are not caused by the polycrystalline nature of the nanosheets. Instead, these are likely caused by dislocations and voids in the nanosheet, some examples of which are indicated by the arrows in Figs. 1a and 2a. The same discontinuities are present in the 300 and 500 °C data but are reduced in magnitude as the total resistance reduces. The 300 and 500 °C data is shown magnified in the inset in Fig. 3.

The regression coefficients are improved after annealing at 300 and 500 °C. Therefore, we attribute the poorer fit of the prior non-annealed case to inconsistent probe-nanosheet contacts due to surface contaminants which are removed during annealing [12]. The regression coefficient at 700 °C decreased due to the partial disintegration of the nanosheet structure as discussed earlier.

**Fig. 4:**
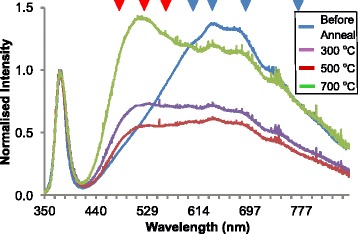
PL spectra of ZnO nanosheets before and after vacuum annealing at 300, 500 and 700 °C with *red arrows* marking the peak positions of p-type defects and *blue arrows* marking the peak positions if n-type defects

PL spectra shown in Fig. 4 are normalised to the near band edge (NBE) peak which remained centred at 378 nm for all annealing stages. Before annealing, the DLE peak maximum is at 640 nm with peak fitting indicating constituent components centred at 595, 635, 690 and 765 nm. These components correspond to transitions from: an oxygen vacancy with a single positive charge to an oxygen interstitial with no charge [22–24]_,_ an oxygen vacancy with a single positive charge to an oxygen vacancy with no charge [25], an oxygen vacancy with no charge to the valance band [26], the conduction band to an oxygen vacancy with no charge [26–28], respectively. These DLE transitions are caused by electron donor defect states indicating that the sheets are n-type [2].

After annealing to 300 °C, the intensity of the DLE peak to decrease relative to the NBE with the components remaining centred at the same positions. However, annealing caused components centred at 470, 520 and 543 nm to increase which are attributed to the p-type defect transitions: an interstitial oxygen ion with a single negative charge [29], an oxygen occupying a zinc site [29, 28, 26] and a transition from the conduction band to an oxygen interstitial with no charge [30, 31, 23, 28]. Further annealing to 500 °C caused the intensity of the DLE peak to decrease relative to the NBE with all components reducing. This is the same effect as seen in previous work when annealing ZnO nanosheets in air at 1000 °C, although annealing in UHV causes the effect to occur at a much lower temperature. Annealing to 700 °C caused all components of the DLE peak to increase relative to the NBE peak. However, the SEM images show that there is significant damage to the ZnO nanosheets; therefore, the PL spectrum for the nanosheets annealed to 700 °C is not reliable.

AES was used to assess the chemical composition of the ZnO nanosheets with annealing.

**Fig. 5:**
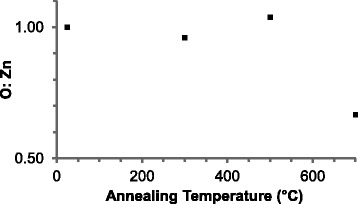
AES of ZnO nanosheets showing percentage of each element before and after annealing compared to silicon

Spectra for the O KLL and Zn LMM transitions were collected from two points on a nanosheet, and two silicon spectra were taken from the substrate at each annealing stage. The average ratio of oxygen and zinc normalised to the average silicon peak intensity is plotted in Fig. 5. This result shows the stoichiometry is retained when annealing up to 500 °C in vacuum. However, annealing at 700 °C caused loss of stoichiometry supporting the SEM image in Fig. 2d that shows the partial disintegrated ZnO nanosheets at the same temperature.

Earlier, the resistance was shown to decrease after annealing at 300 °C, with the data becoming more linear and the gradient reducing. This is caused both by the removal of surface contaminants and the increase in the average grain size reducing the number of grain boundary edges through which electrons must travel. The resistance decrease could have been caused by an increase in n-type defects giving rise to higher apparent doping, although the PL rules this out, instead showing an increase in p-type defects which would in fact compensate for the n-type carriers.

Annealing to 500 °C causes the number of n-type defects to decrease, further reducing the number of charge carriers and causing the observed increase in resistance. Unlike when annealing at 300 °C, at this higher temperature, the additional surface cleaning and sintering effects do not compensate for the reduction in defect doping.

## Conclusions

A low-cost, high throughput microwave method has been used to synthesis ZnO nanosheets using a solution of HMTA and zinc acetate. Vacuum annealing has allowed the study of intrinsic material properties in a controlled environment. Before annealing, the measured resistance against length is less uniform due to surface contamination. At 300 °C, the resistance drops due to surface cleaning and increased grain size, outweighing the reduction in n-type defect doping and the increase in p-type defect doping measured with PL. At 500 °C, the resistance increases as both n-type and p-type defect doping reduces further which is no longer offset by surface cleaning and further grain sintering. Annealing to 700 °C resulted in the partial disintegration of the nanosheet structure, observed both in SEM and by the loss of stoichiometry observed with AES.

Our results are in agreement with previous PL and SEM studies; however, we find that annealing in vacuum causes both the increase in p-type defect formation, and the sintering of the grains, to occur at a significantly lower temperature than observed when annealed in air [14]. Our transport measurements show for the first time that low-temperature annealing of ZnO nanosheets is required prior to contact formation in order to remove surface contaminants and form reliable contacts. Our results also suggest that the operation of any ZnO nanosheet-based device which requires a high-temperature annealing fabrication process step, or is operated at high temperature, will be adversely affected.

## Additional File

Additional file 1: Figure S1. SEM images of probe position a) position one before annealing, b) position two before annealing, c) position three before annealing, d) position four before annealing, e) position five before annealing, f) position one after annealing to 300 °C, g) position two after annealing to 300 °C, h) position three after annealing to 300 °C, i) position four after annealing to 300 °C and j) position five after annealing to 300 °C. (DOCX 358 kb)

## Abbreviations

AES: Auger Electron Spectroscopy
DLE: deep level emission
HMTA: hexamethylenetetramine
LBZA: layered basic zinc acetate
NBE: near band edge
PL: photoluminescence
SEM: scanning electron microscopy
UHV: ultra-high vacuum
