# Supplementary material for: Effects of Vacuum Annealing on the Conduction Characteristics of ZnO Nanosheets
Source: Nanoscale Res Lett. 2015 Sep 17;10:368. doi: 10.1186/s11671-015-1066-1 (PMC4573738; doi:10.1186/s11671-015-1066-1)
Supplement: Additional file 1: Figure S1. — SEM images of probe position a) position one before annealing, b) position two before annealing, c) position three before annealing, d) position four before annealing, e) position five before annealing, f) position one after annealing to 300 °C, g) position two after annealing to 300 °C, h) position three after annealing to 300 °C, i) position four after annealing to 300 °C and j) position five after annealing to 300 °C. (DOCX 358 kb) [file 11671_2015_1066_MOESM1_ESM.docx]

**Supplementary information**

**Figure S1: SEM images of probe position a) position one before annealing, b) position two before annealing, c) position three before annealing, d) position four before annealing, e) position five before annealing, f) position one after annealing to 300 ^o^C, g) position two after annealing to 300 ^o^C, h) position three after annealing to 300 ^o^C, i) position four after annealing to 300 ^o^C and j) position five after annealing to 300 ^o^C**
